# Effects of Language Skills and Strategy Use on Vocabulary Learning Through Lexical Translation and Inferencing

**DOI:** 10.1007/s10936-020-09720-9

**Published:** 2020-07-10

**Authors:** Alaa Alahmadi, Anouschka Foltz

**Affiliations:** 1 School of Languages, Literatures and Linguistics, Bangor University, Bangor, Gwynedd LL57 2DG UK; 2 Department of English Studies, University of Graz, Graz, Austria

**Keywords:** Vocabulary acquisition, Vocabulary learning strategies, Language skills, Lexical inferencing, Lexical translation

## Abstract

This study explores how vocabulary learning strategy usage and skills in the four language domains relate to participants’ increase in vocabulary size and to the learning of specific vocabulary items over a certain period of time. Sixty-one advanced L1 Arabic L2 learners of English read target words in semi-authentic reading materials and were instructed to either guess the meaning from context or consult a dictionary. Pre- and delayed post-tests assessed vocabulary size and knowledge of the target vocabulary items. Results showed that learning through inferencing, but not learning through dictionary use, depended on learners’ familiarity with the particular learning strategy. Additionally, note taking and reading comprehension influenced lexical knowledge and acquisition in complex ways.

## Introduction

Vocabulary acquisition is an important component of communicative competence and is a core element in language mastery (Baharudin and Ismail 2014). Furthermore, learners’ achievement in the language skills listening, speaking, reading and writing relies on their vocabulary knowledge (Richards and Renandya 2002). Weigand (1998, p. 44) argued that “to learn a language means to know how words are used and what utterances are used in specific situations”. The vital role of vocabulary in language learning has triggered a large amount of research (cf. Akpınar et al. 2015; Milton 2009; Nation 2001; Schmitt 2000; Wang 2007).

Despite its important role in language learning, learners often identify vocabulary as the most challenging area. Various explicit and implicit techniques and strategies, so-called vocabulary learning strategies (VLS), have been identified and developed to help learners acquire vocabulary (e.g. Gu and Johnson 1996; Schmitt and Schmitt 2011; Schmitt 1997; Nation 2001). We follow Catalan’s (2003, p. 56) definition of vocabulary learning strategy as:

knowledge about the mechanisms (processes, strategies) used in order to learn vocabulary as well as steps or actions taken by students (a) to find out the meaning of unknown words, (b) to retain them in long-term memory, (c) to recall them at will, and (d) to use them in oral or written mode.

Vocabulary learning strategies form a sub-class of language learning strategies (LLS), which are “the conscious thoughts and actions that learners take in order to achieve a learning goal” (Chamot 2004, p. 14). O’Malley and Chamot (1990) suggest that most LLS could be applied for completing word learning tasks. Several scholars have proposed VLS taxonomies (e.g. Gu and Johnson 1996; Nation 2001; Schmitt 1997). The current study will follow Schmitt’s (1997) VLS taxonomy, which divides VLS into two major classes: (a) discovery strategies, which refer to learners’ attempts to work out the meaning of novel words and (b) consolidation strategies which refer to learners’ ability to solidify initially learned word meanings. We focus on two specific discovery strategies: lexical inferencing and lexical translation. Lexical inferencing refers to using context cues to guess a word’s meaning, whereas lexical translation refers to obtaining the word’s meaning from a language resource, such as a dictionary. Generally, discovery strategies include two sub-categories: determination and social strategies. Determination strategies involve learners’ direct attempts to determine a novel word’s meaning from a limited set of choices, including dictionary consultation and contextual inferencing. Social strategies involve interactions with other speakers. Lexical translation by means of asking a teacher about a word’s meaning would represent a social strategy.

In this study, we use a longitudinal design to investigate how learners’ self-reported use of VLS and self-reported proficiency and language skills relate to their overall vocabulary size as well as the vocabulary learning that occurs over a certain period of time through lexical inferencing (guessing meaning from context) and lexical translation (looking words up in a dictionary).

### Vocabulary Learning Strategies and Vocabulary Size

Previous studies have revealed a statistically significant positive relationship between the use of certain VLS and lexical knowledge (Alahmadi 2015; Alahmadi et al. 2018; Al Qahtani 2005; Alqurashi 2013; Alsaif 2011; Hamzah et al. 2009; Tanyer and Ozturk 2014). Of most relevance for the current study are VLS that relate to guessing the meaning of words from context and to dictionary use. Several studies have found positive relationships between these two VLS and vocabulary knowledge in terms of breadth. For example, Alahmadi (2015), Alahmadi et al. (2018) and Alsaif (2011) found a statistically significant positive relationship between the strategy of *guessing a word’s meaning from the provided context* and English vocabulary knowledge in terms of breadth in Saudi participants across a range of educational levels. More generally, Alqurashi (2013) found that determination strategies, which include *using monoligual or bililingual dictionaries* and *inferring meaning from context*, showed the highest contribution (44%) to learners’ vocabulary size, followed by social strategies (37%).

### Vocabulary Learning Strategies, Inferencing and Lexical Translation

As determination strategies, both guessing from context and obtaining meaning through dictionary use involve conscious attempts to determine word meaning from a limited set of choices. It seems that such conscious attempts would require some skills on the part of the learner. For instance, Haastrup (1991, p. 39) defined inferencing as the ability to use “all available linguistic cues in combination with the learner’s general knowledge of the world, her awareness of the co-text and her relevant linguistic knowledge”. In order to engage in lexical inferencing successfully, learners would need to have all of the above-mentioned knowledge and abilities. Similarly, dictionary use requires learners to find the appropriate entry in a dictionary and then select the translation that is appropriate in the particular context from what is typically a variety of translation choices. Thus, experience with inferring meaning from context or with using a dictionary may relate to learning new vocabulary more successfully when using these strategies.

Fraser (1999) investigated three vocabulary learning strategies (ignore, consult, infer) and their effect on participants’ vocabulary attainment and found that different strategies have different levels of success. While lexical inferencing was participants’ first choice to determine a novel word’s meaning, dictionary consultation had a higher success rate (78%) than lexical inferencing (52%).

Marefat and Shirazi (2003) found that directly teaching vocabulary learning strategies impacted participants’ short- and long-term vocabulary retention. Their results for short-term retention showed that lexical translation led to similar retention (44%) compared to using linguistic clues for inferencing (45%), with using non-linguistic clues for inferencing having somewhat higher retention rates (56%). Their long-term retention results showed rather low retention rates for lexical translation (28%), with somewhat higher rates for linguistic inferencing (37%) and yet higher rates for non-linguistic inferencing (51%).

### Language Skills and Vocabulary Size

Various researchers have noted the influence of lexical knowledge on the four language skills (listening, speaking, reading and writing). Most of these studies focus on reading skills (e.g. Laufer 1992; Ouellette 2006; Qian 1999, 2002). Schmitt et al. (2011, p. 39) argue that “there is a fairly straightforward linear relationship between growth in vocabulary knowledge for a text and comprehension of that text”. In line with this, Stæhr (2008) found a stronger relationship between vocabulary size and reading skills than vocabulary size and writing or listening skills. While all three skills produced statistically significant correlations with learners’ vocabulary size in terms of breadth, reading skills produced the highest (0.83) and listening the smallest (0.69) correlation. Similarly, a regression analysis showed that vocabulary size accounted for 72%, 52% and 39%, respectively, of the variance in learners’ ability to score above the mean in reading, writing and listening tests.

In terms of speaking skills, Koizumi and In’nami (2013) concluded that vocabulary knowledge both in terms of breadth and depth plays a significant role in learners’ speaking proficiency. Specifically, they found significant relationships between vocabulary knowledge and speaking skills. Across two studies, learners’ vocabulary knowledge accounted for 44% and 84%, respectively, of participants’ speaking proficiency. Furthermore, 63% and 60%, respectively, of learners’ speaking proficiency could be accounted for by breadth and depth of vocabulary knowledge alone.

In line with such results, some reseachers have proposed a minimum level of vocabulary size needed for certain language tasks. Milton (2009), for instance, suggested a vocabulary size of 3000 words to successfully engage in a simple conversation. Laufer (1989) proposed a threshold of 5000 word families for an average of 95% text coverage for academic texts. Similarly, Laufer and Ravenhorst-Kalovski (2010) and Nation (2006) proposed a level of 8000 word families for 98% text coverage for a variety of authentic texts.

### Language Skills, Inferencing and Lexical Translation

Studies on the relationship between language skills and the strategies of lexical inferencing and lexical translation have been inconclusive. While Bensoussan and Laufer (1984) found no correlation between learners’ ability to infer meaning correctly and their language skills, measured as proficiency level, Haynes (1984) found a significant effect of language proficiency level on successfully determining appropriate meanings through inferencing, with learners with higher proficiency levels successfully guessing more of the target words than learners with lower proficiency levels. Similarly, Knight (1994) found an effect of dictionary consultation on reading comprehension, such that learners who consulted a dictionary did not only learn more words, but also achieved higher reading comprehension levels.

## Current Study

The current study follows on from the results of Alahmadi (2015) and Alahmadi et al. (2018), where we found a significant positive relationship between inferencing and vocabulary size across two participant groups. However, it was not clear whether engaging in inferencing increased participants’ vocabulary size or whether learners with larger vocabulary sizes found inferencing easier (de la Garza and Harris 2017) and therefore used it more frequently. The current study explores this issue through a longitudinal design. It also follows on from Alahmadi and Foltz’s (under revision) longitudinal results, which found similar levels of vocabulary learning for both lexical inferencing and lexical translation as well as a significant influence of learners’ overall vocabulary size on the amount of learning that occurred when engaging in inferencing and dictionary use. Specifically, learners with larger vocabulary sizes learned more lexical items through both inferencing and dictionary use over the course of the study than learners with smaller vocabulary sizes. Here, we expand on this previous work and explore whether language skills or familiarity with learning strategies involving guessing or dictionary use also influence the amount of learning that occurs when engaging in inferencing and dictionary use.

Participants in the current study learned target words in authentic reading materials during two training phases. They were asked to guess some of the target words from context and look up others in a dictionary. Their vocabulary size in terms of breadth was measured before and after training. In addition, an English-language self-assessment questionnaire assessed learners’ proficiency level and language skills and a VLS questionnaire assessed participants’ VLS usage.

In this paper, we expand our previous results by considering the information from the VLS and English-language self-assessment questionnaires, which was beyond the scope of Alahmadi and Foltz (under revision). Specifically, we investigate how VLS usage and language skills in the four domains relate to participants’ increase in vocabulary size and to how many words participants learned through guessing and dictionary use over the course of the study. The current study aims to answer the following research questions (RQs):

- RQ1: Is participants’ VLS usage related to their vocabulary size in general and to the increase in their vocabulary knowledge over the course of the study?
- RQ2: Is participants’ VLS usage related to how well they learn the words through guessing or dictionary use throughout the duration of the study?
- RQ3: Are participants’ self-assessed English language skills related to their vocabulary size in general and to the increase in their vocabulary knowledge over the course of the study?
- RQ4: Are participants’ self-assessed English language skills related to how well they learn the words through guessing or dictionary use throughout the duration of the study?

## Methodology

### Participants

The study comprised 61 senior undergraduate Saudi English-major students [47 (77%) males and 14 (32%) females] from three Saudi Universities. Informed consent was obtained from all participants included in the study. Participants’ ages varied from 20 to 28 years (mean = 22.75; *SD* = 1.626). They were all native speakers of Arabic who started learning English in grade 4 of primary school. They received an approximate number of 1600 h of EFL tuition between their public school and university education (Alqurashi 2013).

### Materials and Procedures

The study involved three phases: a pre-test, two training sessions, and a delayed post-test. The following sections describe the materials and procedures for each phase. Data and analysis scripts are available at https://osf.io/hd4rp/.

### Pre-test Materials

#### English Language Self-Assessment Questionnaire

An English language self-assessment questionnaire, given in participants’ L1 Arabic to avoid that proficiency level interfered with responses, assessed participants’ English language skills and usage. Following questions about basic demographic information, the questionnaire was divided into five sections. In the first section, participants rated their English proficiency level by ticking one of the options beginner (0), intermediate (1), advanced (2), fluent (3) or near native (4). Moreover, they rated their English use outside of the classroom on a Likert scale from always (4), frequently (3), sometimes (2), rarely (1) to never (0). The remaining four sections assessed the individual language skills (listening, speaking, reading and writing), with three questions per section. Participants evaluated how commonly different statements regarding each language skill applied to them. For instance, in the listening section, participants replied to statements like *I can easily follow lectures and presentations when they are conveyed clearly*, applying the Likert scale mentioned above.

#### Word Translation Task

A word translation task gauged learners’ knowledge of the target words used during the training phase. Participants translated 24 target and 24 control words. The target words occurred in the training sessions, whereas the control words did not (see the section on reading texts below). Target and control words were matched for frequency, difficulty, word length, derivational complexity and part of speech, which did not differ significantly across target and control words (all *p* > 0.1; see Alahmadi and Foltz, under revision, for details). Difficulty level was assessed through a norming study with 16 senior undergraduate English major Saudi students (11 females, 5 males; mean age = 22.38, *SD* = 3.03; self-assessed proficiency level of 2.43, *SD* = 0.72). Students translated the target and control words from English into Arabic without the use of any translation aids. An average of 2.40 (*SD* = 1.86) target words and 2.13 (*SD* = 2.06) control words were translated correctly, again a non-significant difference (generalized linear model with family = “poisson”: estimate = 0.1, SE = 0.19, *z* = 0.62, *p* = 0.53). Thus, target and control words had similar frequencies, word lengths, derivational complexity, parts of speech, and difficulty levels. Moreover, difficulty levels were sufficiently high to allow for learning, with participants in the norming study correctly translating only a minority of both target and control words.

#### The XK_Lex Vocabulary Size Test

Participants’ lexical knowledge was calculated using the XK_Lex vocabulary breadth size test designed by Al-Masrai and Milton (2012). The test estimates EFL/ESL learners’ vocabulary knowledge in terms of breadth out of the most frequent 10,000 words in English. XK_Lex is a reliable and valid vocabulary breadth test (Al-Masrai 2009). In this paper-and-pencil checklist test, participants check all the English words that they know. To reduce the amount of guessing, the test includes 100 real words and 20 pseudo words. Learners’ vocabulary size in terms of breadth is calculated by adding up all the checked real words and multiplying the result with 100, then adding up all the checked pseudo words and multiplying the result with 500, then subtracting the latter product from the first.

### Pre-test Procedures

Participants were tested during their normal class sessions. After giving informed consent, which included access to students’ academic Grade Point Average (GPA), participants completed the English language self-assessment questionnaire, the word translation task, and finally the XK_Lex vocabulary breadth size test (cf. Alahmadi and Foltz, under revision, for more detailed information).

### Training Materials

#### Reading Texts

Four texts from Schmitt and Schmitt’s (2011) and de Chazal and Rogers’ (2013) textbooks for English learners were adapted for the reading comprehension task used during training. All texts were of medium difficulty in terms of vocabulary and had similar lengths. Each text contained six of the 24 target words from the word translation task described above. These words were underlined in the texts. Participants translated the target words that they knew in one column, and the target words that they did not know in another. To translate words that they did not know, participants were either instructed to guess their meaning from context or to look them up in a dictionary. Two multiple-choice questions following each text assessed participants’ text comprehension (cf. Alahmadi and Foltz, under revision, for further details).

### Training Procedures

The training sessions occurred two and three weeks, respectively, after the pre-test. Prior to training, participants were distributed into a low and high proficiency group based on their GPA, their vocabulary size, and the word translation task. Participants whose scores across two of the measures were above the median for these measures were considered to have high proficiency, those whose scores were below the median for two measures were grouped as low proficiency. Based on this, participants were distributed across two training groups, such that half the participants in each group were of low, and the other half of high proficiency. This was done to ensure a similar spread of proficiencies across groups. During each training session, participants read two of the texts and completed the associated tasks. Both training groups completed the same tasks (guessing vs. dictionary look-up) in the same order, but the texts were counter-balanced across training groups, such that for each particular text, one group engaged in guessing and the other in dictionary look-up.

### Coding of Responses

The first author scored participants’ translations of the target words using Wesche and Paribakht’s (2009) scoring system. Each semantically and syntactically appropriate translation received one point. Any semantically, but not syntactically, appropriate translation received half a point. Incorrect translations received no points. A second Arabic-English bilingual additionally scored translations from 20 randomly-selected participants, with high inter-coder agreement (Cohen’s Kappa κ = 0.987; *p* < 0.001).

### Delayed Post-test Materials

#### Word Translation Task and the XK_Lex Vocabulary Size Test

Participants again completed the word translation task and the XK_Lex vocabulary size test.

#### VLS Questionnaire

Participants completed a vocabulary learning strategies (VLS) questionnaire that gauged their VLS usage, again provided in their L1 Arabic to avoid that proficiency level interfered with their responses. Ten VLS items were included, based on Ahmed (1988), Al Qahtani (2005), Alsaif (2011), O’Malley and Chamot (1990) and Oxford (1990). In this paper, we will focus on the eight VLS that relate to lexical inferencing and lexical translation. The questionnaire used the above-mentioned Likert scale from always (4) to never (0).

### Delayed Post-test Procedures

Participants completed the delayed post-test two weeks after the training sessions. Participants first completed the word translation task, then the XK_Lex vocabulary size test, and finally the VLS questionnaire.

## Results

### Participant Profiles

Before addressing the research questions, we will provide a profile of the participants by summarizing their self-rated proficiency and language use, their vocabulary size, and their responses to the questions in the VLS questionnaire (Table 1) and the English-language self-assessment questionnaire (Table 2). Participants’ average self-rated English proficiency was 2.31 (*SD* = 0.71) on a scale from *beginner* (0) to *near*-*native* (4), which represents a score between *advanced* (2) and *fluent* (3). On average, participants used English outside of the classroom only *sometimes* (2), with an average rating of 1.96 (*SD* = 0.98). Participants’ average vocabulary size during the pre-test was 3331 words (*SD* = 1318), which increased to 3837 words (*SD* = 1400) after the training sessions. Both average vocabulary sizes are somewhat lower than what has been proposed for high text coverage and effective usage of inferencing strategies (Laufer and Ravenhorst-Kalovski 2010). Also notice that the increase in vocabulary size with an average of 506 lemmas over the five weeks of the study seems rather large. This number is surely somewhat inflated because participants took the same XK_Lex vocabulary size test twice only five weeks apart. However, the overall magnitude of the increase is compatible with some previous studies (Webb 1962; Cobb and Horst 2001). For example, based on Brysbaert et al.’s (2016) estimates, Laufer (1998) found that 11th graders had passively learned about 500 lemmas a month.

**Table 1:** Participants’ mean frequency ratings for the assessed VLS with ratings from *frequently* (4) to *never* (0)

VLS statement	Mean (*SD*)
I use a traditional English/Arabic dictionary to find out the meaning of a new word	2.85 (0.85)
I underline the word and use a special application in my phone to find out the meaning	2.59 (0.95)
I try to infer the right meaning of this word from its context	2.45 (0.86)
I apply the grammar cues strategy to infer the meaning of novel words, for instance, -*ment* or *-tion* = noun	2.40 (1.10)
I enquire with my instructor about the meaning of the novel word	2.24 (1.13)
I consult a fellow student about the new word’s meaning	2.18 (1.11)
I try to write the new word in a full sentence	1.90 (1.22)
I use a traditional English/English dictionary to find out the meaning of a new word	1.68 (1.28)

*SD* standard deviation

**Table 2:** Participants’ mean frequency ratings in the English language self-assessment questionnaire with ratings from *frequently* (4) to *never* (0)

Skill	Questionnaire statement	Mean (*SD*)
Reading	I recognise the main ideas when reading texts in my course textbooks	2.76 (0.99)
	I can locate the information that I need in a general text in a quick and easy manner	2.65 (0.88)
	I can comfortably read complex lengthy texts, stories and articles	1.84 (1.19)
Writing	I can take notes during lectures	2.49 (1.02)
	I can freely write my opinion on a variety of topics	2.28 (0.94)
	I can build up my arguments in a logical way within an essay	1.61 (1.15)
Listening	I can easily follow lectures and presentations when they are conveyed clearly	2.90 (0.81)
	I can understand informal conversations on common topics	2.76 (0.92)
	I can understand the news on the radio or TV	2.34 (0.98)
Speaking	I can express myself confidently within informal life situations	2.68 (0.85)
	I can participate in an academic argument during lectures	2.11 (1.06)
	I can present an academic topic in front of my class	2.03 (1.24)

*SD* standard deviation

Table 1 shows that participants are moderate users of VLS, as most of their mean frequency ratings for the provided statements are between *sometimes* (2) and *frequently* (3). Bilingual dictionary use is the most commonly used strategy with a mean value of 2.85 (*SD* = 0.85), whereas monolingual dictionary use is the least commonly used strategy with a mean value of 1.68 (*SD* = 1.28). Lexical inferencing use is between these values, with an average rating of 2.45 (*SD* = 0.86).

Table 2 shows that most of participants’ ratings in the English language self-assessment questionnaire are between *sometimes* (2) and *frequently* (3), suggesting that they can achieve the tasks mentioned in the questionnaire moderately frequently. Following clearly conveyed lectures and presentations received the highest mean rating (2.9), indicating that participants are frequently able to do so. In contrast, essay writing represents a difficulty with a mean rating (1.61) between *rarely* (1) and *sometimes* (2).

### VLS and Breadth of Vocabulary Knowledge (RQ1)

We first examined whether there is a relationship between participants’ reported VLS usage and their vocabulary size prior to the training sessions. To explore this, we used a generalized linear model (GLM). Participants’ vocabulary size according to the pre-test was the dependent variable and ratings for all VLS were the independent variables. All independent variables were centred before analysis to minimize collinearity. The independent variables that did not significantly contribute to model fit were removed from the models in a step-wise procedure to yield the final statistical models (cf. Baayen 2008). There was a statistically significant main effect of asking the instructor about a word’s meaning on pre-test vocabulary size (estimate = − 407.3; SE = 133.3; *t* = − 3.06; *p* = 0.003), showing that students with lower vocabulary sizes used this strategy more often than students with higher vocabulary sizes. Second, there was a statistically significant main effect of inferencing meaning from context on participants’ vocabulary size highlighting that learners with greater vocabulary sizes engaged in lexical inferencing more often than their counterparts with lower vocabulary sizes (estimate = 676.8; SE = 174.5; *t* = 3.88; *p* < 0.001).

We also examined whether there is a relationship between participants’ self-reported VLS usage and their gain in vocabulary size over the duration of the study. The generalized linear model had gain in vocabulary size (participants’ vocabulary size in the delayed post-test minus their vocabulary size in the pre-test) as the dependent variable and ratings for all VLS as independent variables. Again, all independent variables were centred prior to analysis and removed from the model if they did not contribute to model fit. The final model revealed a significant main effect of bilingual dictionary use on overall vocabulary gain (estimate = 333.4; SE = 114.6; *t* = 2.91; *p* = 0.005), indicating that learners who reported using a bilingual dictionary more often increased their vocabulary knowledge more over the course of the study than learners who reported using a bilingual dictionary less often.

### VLS and Retention of Inferred and Looked Up Words (RQ2)

We then investigated whether participants’ VLS usage (independent variables) impacted their amount of vocabulary learning for words that they were instructed to infer or look up in a dictionary (dependent variables) during the training sessions. We conducted two separate analyses, one for learning through inferencing and one for learning through dictionary use, using the same procedures and model comparisons as before. The final model for the inferencing condition revealed a significant main effect of guessing from context on learning words through inferencing during training (estimate = 0.197; SE = 0.086; *t* = 2.28; *p* = 0.026), such that participants who reported using the lexical inferencing from context strategy more often learned more words when asked to engage in inferencing during the study than participants who reported using this strategy less regularly. The final model for the dictionary condition had no fixed effects. In other words, none of the factors that we looked at contributed to model fit.

### Self-Assessment Questionnaire and Vocabulary Knowledge (RQ3)

Next, we explored the relationship between how participants rated themselves in the self-assessment questionnaire and their breadth of vocabulary knowledge prior to the two training sessions. Participants’ responses to questions in the self-assessment questionnaire were the independent variables and their vocabulary knowledge according to the pre-test was the dependent variable in the generalized linear models. The analysis procedure was the same as before. The final model produced two significant main effects. First, we found a significant main effect of the self-reported ability to recognize the main ideas when reading texts on vocabulary size prior to training (estimate = 441.7; SE = 167.9; *t* = 2.63; *p* = 0.011). Participants with higher vocabulary sizes prior to the study reported recognising the main ideas when reading texts more often than participants with lower vocabulary sizes. Second, we found a significant main effect of the strategy of taking notes during lectures on participants’ vocabulary size prior to training (estimate = 331.7; SE = 158.7; *t* = 2.09; *p* = 0.041). Participants with higher vocabulary sizes reported taking notes during lectures more often than participants with lower vocabulary sizes.

We also examined whether there is a relationship between how students rated themselves in the self-assessment questionnaire and the increase of their vocabulary size over the course of the study. Generalized linear models included responses to the statements of the self-assessment questionnaire as independent variables and learners’ vocabulary size gain during the study, i.e. delayed post-test vocabulary size minus pre-test vocabulary size, as dependent variable. The analysis procedure was the same as above. The final model showed two significant main effects. First, there was a significant main effect of the strategy of taking notes during lectures on participants’ overall vocabulary growth over the duration of the study (estimate = − 245.2; SE = 100.5; *t* = − 2.44; *p* = 0.018). Surprisingly, participants who reported being able to take notes less often improved their overall vocabulary more than participants who reported being able to take notes more frequently. There was also a significant main effect of presenting an academic topic on learners’ overall vocabulary size increase over the course of the study (estimate = 191.6; SE = 84.77; *t* = 2.26; *p* = 0.028). Specifically, participants who reported being able to present an academic topic more often increased their vocabulary size more over the course of the study than participants who reported being able to present an academic topic less often.

### Self-Assessment Questionnaire and Retention of Inferred and Looked-Up Words (RQ4)

Finally, two GLMs tested whether there is a relationship between how students rated themselves in the self-assessment questionnaire (independent variables) and their retention level for words that they were instructed to guess or look up (dependent variables) during the training sessions. Again, the same analysis procedure as above was used. The analysis for the guessing from context condition revealed no significant main effects. None of the independent variables that we looked at contributed to model fit. The analysis for the dictionary condition revealed two significant main effects. First, we found a significant main effect of how participants rated themselves in the skill of finding needed information in a general text on the size of their learning effect through dictionary use (estimate = 0.184; SE = 0.066; *t* = 2.81; *p* = 0.007). In particular, participants who reported more frequently being able to find needed information in a general text retained more of the words that they looked up in a dictionary as part of the training sessions than participants who reported less frequently being able to find needed information in a general text. Second, the results revealed a significant main effect of how students rated themselves in the skill of freely writing their opinions on the size of their learning effect through dictionary use (estimate = − 0.150; SE = 0.062; *t* = − 2.43; *p* = 0.018). Interestingly, participants who reported less commonly being able to freely write their opinions learned more of the words that they looked up in a dictionary than participants who reported more often being able to freely write their opinions.

## Discussion

The current research explored how VLS usage and language skills relate to vocabulary size and vocabulary learning over a certain period of time. In the following sections, we will summarize the results and discuss them with respect to our four research questions.

### VLS Usage and Vocabulary Size (RQ1)

The first research question explored the potential relationship between participants’ VLS usage and their pre-test vocabulary size as well as their increase in vocabulary size over the course of the study. We found that (1) participants with lower vocabulary sizes reported asking instructors about word meanings more frequently than those with higher vocabulary sizes. In addition, (2) participants with larger vocabulary sizes reported engaging in more inferencing from context than those with smaller vocabulary sizes. Furthermore, (3) participants who reported using a bilingual dictionary more often increased their vocabulary size more over the course of the study than participants with less bilingual dictionary usage.

The first finding is inconsistent with Alahmadi et al. (2018) who found no such effect for postgraduates and the reverse effect for undergraduates, namely that those with *higher* vocabulary sizes reported asking instructors about word meanings more frequently than those with *lower* vocabulary sizes. This discrepancy may be due to participants’ different vocabulary sizes across the two studies. Undergraduates in Alahmadi et al. (2018) had an average vocabulary size of 1976 words, compared to 3331 words for the current undergraduate participants and 5368 words for Alahmadi et al. (2018)’s postgraduates. This pattern of results from Alahmadi et al. (2018) and the current study could be explained by an inverse U-shaped relationship between vocabulary size and asking instructors about word meanings. Specifically, among the group with the lowest vocabulary sizes, use of this VLS increases as vocabulary size increases, possibly as learners become more confident in asking questions. Among the group with medium vocabulary sizes, use of this VLS decreases as vocabulary size increases, possibly because learners have less need to ask their instructor about words’ meanings as their vocabulary size increases. No effect for this VLS was found for the group with the highest vocabulary size, who used this strategy very infrequently overall. This suggests that learners may make use of this strategy more often at a certain stage in their learning that corresponds to a particular vocabulary size.

The second finding is consistent with various previous studies, such as Alahmadi et al. (2018), Alsaif (2011) and Al Qahtani (2005), who found a significant positive relationship between participants’ inferencing strategy use and their vocabulary size for learners of various proficiency levels. However, the current results contradict Alqurashi (2013), who found no relationship between inferencing strategy use and vocabulary size. As we have argued in Alahmadi et al. (2018), it is not clear whether learners with higher vocabulary sizes choose to engage in inferencing more frequently, possibly because they know more words in the immediate context, which facilitates engaging in inferencing (de la Garza and Harris 2017), or whether learners who engage more frequently in inferencing increase their vocabulary size as a result of this relatively deep engagement with the text (cf. Richards 1976).

The third finding is consistent with studies that find a positive relationship between bilingual dictionary use or determination strategies more generally and vocabulary size (Hamzah et al. 2009; Komol and Sripetpun 2011). The current study goes beyond these previous results in showing that frequent self-reported use of a bilingual dictionary contributed to learners’ vocabulary size increase over a certain period of time. Our results support Kroll and Curley’s (1988) claim that using L1 equivalents to learn novel L2 words is an efficient vocabulary acquisition method. Specifically, dictionaries are easily accessible (e.g. in phone applications) and allow viewing examples, synonyms or antonyms for the relevant lexical items so that an orthographic and aural representation for the target word can be acquired. In contrast, we found no evidence for Hamzah et al.’s (2009) claim that the relationship between bilingual dictionary use and vocabulary size in previous studies is due to learners’ inadequate knowledge of other VLS. Participants in the current study engaged in various other VLS with mean values between *sometimes* (2) and *frequently* (3).

### VLS Usage and Retention of Inferred and Translated Words (RQ2)

The second research question investigated the potential relationship between participants’ VLS usage and the amount of vocabulary learning that occurred through guessing and dictionary look-up throughout the study. We found that learners who reportedly use the strategy of guessing from context more often overall learned more vocabulary items through inferencing from context than those who use the strategy of guessing from context less often. In contrast, we found no effect of any VLS on learning through dictionary use. Our results suggest that how successfully learners can acquire vocabulary through inferencing may be due to how familiar they are with inferencing as a strategy. No such familiarity effect was found for dictionary use. Together with the results for RQ1, this paints a picture of both dictionary use and inferencing contributing to vocabulary acquisition, with only inferencing being a strategy whose success seems to depend on having practice with the strategy.

Our results are consistent with Nassaji (2003) who differentiates between strategy use and learners’ ability to apply strategies in an appropriate and effective manner. Specifically, our results suggest that, for some strategies, frequent strategy use relates to success in using the strategy. Our results are also consistent with Hulstijn (1992) who argued that inferencing can support comprehension and learners’ short- and long-term lexical retention. Marefat and Shirazi (2003) highlighted a similar effect of non-linguistic inferencing on learners’ short- and long-term retention level. With regards to explicit instruction of inferencing skills, both Fraser (1999) and Marefat and Shirazi (2003) found no direct effect of explicit instruction on vocabulary acquisition, but noted an indirect influence, such that better inferencing skills related to learners ignoring fewer unfamiliar words. Furthermore, Mondria (2003) found that direct instruction of inferencing strategies had a positive impact on learners’ retention level, but was less efficient in terms of time. Our results do not speak directly to explicit instruction, as participants in the current study were not explicitly taught inferencing or lexical translation strategies, but it does suggest that familiarity with inferencing, which could be achieved through explicit instruction (as, for example, in Alyami and Mohsen 2019), supports learning vocabulary through inferencing.

### Language Skills and Breadth of Lexical Knowledge (RQ3)

The third research question explored the potential relationship between participants’ language skills and their pre-test vocabulary size as well as their increase in vocabulary size over the course of the study. We found that participants who self-reported more often (1) being able to recognize the main ideas when reading texts and (2) taking notes during lectures had higher vocabulary sizes prior to training than participants who self-reported doing so less often. In addition, we found that (3) participants who self-reported more often taking notes during lectures increased their vocabulary size less over the course of the study than participants who self-reported doing so less often. Finally, (4) participants with higher ability to present an academic topic increased their vocabulary size more during the study than participants with lower ability to present an academic topic.

The first result is consistent with numerous previous studies (e.g. Al-Nujaidi 2003; Schmitt and Schmitt 2011; Laufer 1992; Qian 1999, 2002) that found a positive relationship between vocabulary size and reading comprehension, and supports the common argument that sufficient vocabulary knowledge is needed for adequate text comprehension. The second result finds mixed support in the previous literature. While Hamzah et al. (2009) and the current study found a positive relationship between note taking and vocabulary size, Komol and Sripetpun (2011) found no such relationship. Interestingly, our third result finds that learners who engaged in note taking more regularly increased their vocabulary size less over the course of the study than participants who reported using this strategy less frequently. This finding, however, does not necessarily contradict our second result above. It seems that participants who frequently engaged in note taking had higher vocabulary sizes to begin with and thus less opportunity to increase their vocabulary size over the course of the study by means of the intermediate-level texts used during training. This view is consistent with Alahmadi’s (2015) observation that some VLS did not seem to influence a learner’s vocabulary knowledge when the student had reached a certain level of vocabulary size.

The fourth result is consistent with Koizumi and In’nami’s (2013) finding of a significant relationship between learners’ vocabulary knowledge and their ability to express themselves fluently. Adolphs and Schmitt (2003) also noted the role of vocabulary size on influencing speaking ability when they concluded that “more vocabulary is necessary in order to engage in everyday spoken discourse than was previously thought. The implication is that a greater emphasis on vocabulary development is necessary as part of aural improvement” (p. 425). However, while these previous studies suggest a relationship between speaking ability and vocabulary size, our results suggest a relationship between the ability to talk about an academic topic and vocabulary learning. One possibility is that learners with superior presentation skills also have a higher willingness to speak (Heidari 2019) and thus engaged in more conversations in the L2 over the course of the study, which could have contributed to their larger increase in vocabulary knowledge. However, additional correlation analyses suggest that this is unlikely. The current study finds neither a significant correlation between the ability of talking about an academic topic and using English outside of the classroom (*t* = 1.17, *df* = 59, *p* = 0.25) nor between using English outside of the classroom and an increase in vocabulary size (*t* = − 1.49, *df* = 59 *p* = 0.14).

### Language Skills and Retaining Inferred and Translated Words (RQ4)

The fourth research question investigated the potential relationship between participants’ language skills and the amount of vocabulary learning that occurred through guessing and dictionary look-up throughout the study. None of the self-rated language skills related significantly to how many of the target words participants learned during the training sessions through inferencing. For learning target words through dictionary use, we found that (1) participants who more frequently found needed information in a general text learned more of the target words through dictionary look-up than participants who report being able to do so less often. In addition, (2) participants who reported to more often being able to freely write their opinions learned fewer target words through dictionary use than participants who reported being able to do so less often. The first result is consistent with Knight (1994) who found a positive relationship between reading comprehension and dictionary consultation. It is possible that learners who are good at finding information in texts are also good at finding appropriate translations when using a dictionary. The second finding is more puzzling. It seems reasonable that one’s ability to freely write one’s opinions may be unrelated to vocabulary acquisition through dictionary use, but it is not immediately clear how writing ability should relate to less learning through dictionary use. More studies are needed to see if this effect can be confirmed and, if so, what may be causing it.

## Conclusion

The current results highlight that vocabulary acquisition through inferencing, but not through dictionary look-up, depends on learners’ familiarity with this strategy. Interventions that familiarize learners with inferencing strategies may therefore positively impact word learning in a foreign language. Furthermore, reading comprehension and note taking seem to relate to vocabulary size and vocabulary acquisition in complex ways. Overall, we suggest that familiarity with inferencing strategies can benefit learners and that the relationship between note taking and vocabulary acquisition warrants further investigation.
